# Association Between Vasomotor Symptoms of Menopause and Retirement: A Prospective Longitudinal Study

**DOI:** 10.1111/1471-0528.70264

**Published:** 2026-05-12

**Authors:** Biresaw Wassihun Alemu, Michael Waller, Leigh R. Tooth

**Affiliations:** ^1^ Australian Women and Girl's Health Research Centre, School of Public Health, Faculty of Health, Medicine and Behavioural Sciences The University of Queensland Brisbane QLD Australia

**Keywords:** midlife women, retirement, vasomotor symptoms, workforce participation

## Abstract

**Objective:**

To assess the association between vasomotor symptoms of menopause and retirement in Australian women.

**Design:**

Population‐based cohort study.

**Setting:**

Secondary analysis of six surveys collected between 1996 and 2010.

**Population:**

A total of 6928 Australian women, born between 1946 and 1951.

**Methods:**

Data were obtained from participants in the cohort born between 1946 and 1951 of the Australian Longitudinal Study on Women's Health. Between 1996 and 2010, self‐reported longitudinal survey data were collected. Vasomotor symptoms, specifically hot flushes and night sweats, and retirement status were captured. Data were analysed by discrete time survival analyses using logistic regression.

**Main Outcome Measures:**

Odds of retirement.

**Results:**

In fully adjusted analyses, women who experienced both hot flushes and night sweats reported higher odds of retirement compared to women with no vasomotor symptoms (Adjusted Odds Ratio (AOR) = 1.13, 95% CI: 1.03, 1.25). There was no significant association with retirement for women who experienced only hot flushes (AOR = 0.97, 95% CI: 0.85, 1.11) or only night sweats (AOR = 1.19, 95% CI: 0.94, 1.50), compared with those who experienced no symptoms.

**Conclusions:**

Combined menopausal vasomotor symptoms may contribute to women's retirement from the workforce. Improving access to effective symptom management, along with examining how workplace conditions shape women's experiences of menopause, may help guide strategies to support continued workforce participation in midlife.

## 1 Introduction

Menopause is a key biological milestone [1]. It marks the end of a biological woman's reproductive years, primarily due to the depletion of ovarian follicles [1, 2]. In high‐income countries, the average age of natural menopause is 51 years, whereas it is slightly younger in low‐ and middle‐income countries [2]. Some women may experience premature or early onset of menopause due to genetic factors, medical conditions, or surgical interventions [2].

Vasomotor symptoms (VMS), including hot flushes and night sweats, are common among midlife women [3]. Up to 80% of women experience VMS [4], which often intensify during the perimenopause [5]. Factors such as body mass index, stress, smoking, obesity, diabetes, and lower socioeconomic status have been associated with increased severity of VMS [6, 7, 8]. VMS are linked to metabolic diseases, reduced quality of life and productivity [9, 10, 11].

VMS can hinder women's workforce participation [12]. They can disrupt professional life and affect work attendance [13, 14]. Cross‐sectional studies in the USA and Australia found that VMS are associated with increased work absenteeism and reduced work ability [13, 15]. Likewise, a longitudinal study conducted in the UK revealed that women who experienced VMS and other menopausal symptoms reported higher odds of exiting employment [16].

Retirement is the phase of life in which an individual permanently leaves the workforce [17], often as a result of push and pull factors [18]. Push factors such as ill health, and workplace stress, lead to early retirement [19]. In contrast, pull factors like desire to engage in leisure activities can lead to planned retirement [20]. Reproductive health, particularly the menopause and its associated symptoms are an overlooked factor in midlife women's retirement decisions. A cross‐sectional study found that menopausal women were more likely to retire than other women [21]. In line with this, another study reported that experiencing severe hot flushes in the workplace increase women's intentions to exit the workforce [22].

Despite evidence suggesting an association between VMS and women's work outcomes, several important gaps remain. First, many studies are cross‐sectional [13, 14, 22, 23]. Second, the link between VMS and retirement has been unexplored in longitudinal studies. Only one study has investigated the link between menopausal symptoms and exiting employment [16], but it combined 20 menopausal symptoms into a single composite measure, which limits the ability to determine the independent effect of VMS. VMS are among the most reported symptoms experienced by midlife women, yet their potential association with retirement remains under‐recognised in workplace research [14, 24]. This study aims to address these gaps by investigating the association between VMS and retirement using longitudinal data and will also examine the role of other covariates in this association.

## 2 Methods

### 2.1 Participants

The Australian Longitudinal Study on Women's Health (ALSWH) is a nationwide, community‐based longitudinal study that explores various dimensions of women's health, including healthcare usage and overall well‐being, across different age groups in Australia. Since 1996, ALSWH has been collecting surveys from women in three age cohorts: those born between 1921 and 1926, who were aged 70 to 75 in 1996; those born between 1946 and 1951 (45 to 50 years in 1996); and those born between 1973 and 1978 (18 to 23 years in 1996). The original three cohorts were randomly selected from the national health insurance database (Medicare). A fourth cohort of women born in 1989–1995 was recruited in 2012–2013 via open recruitment methods including the internet and social networking sites. Further information regarding ALSWH methodology can be found in Dobson et al. [25] and Loxton et al. [26].

**FIGURE 1:**
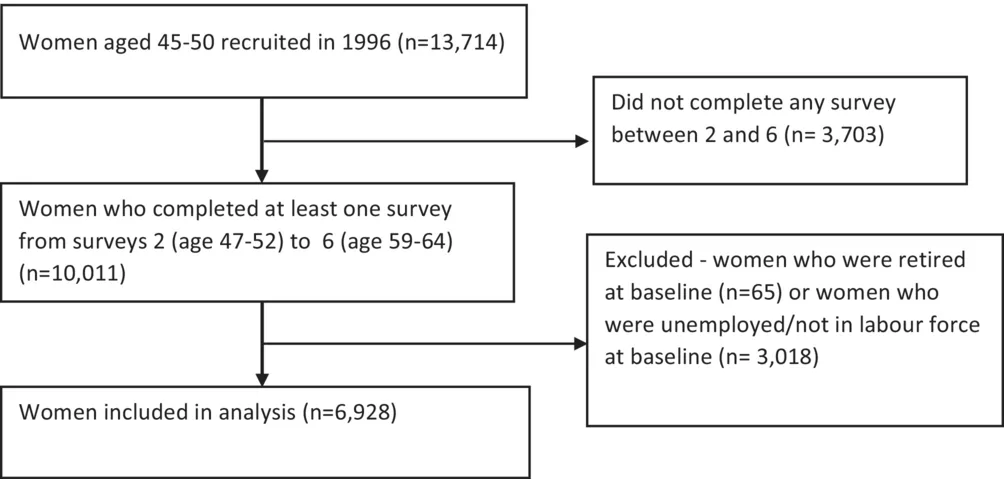
Flowchart showing the process of selecting the sample from the 1946–1951 ALSWH cohort.

For this study we used data from the 1946–1951 cohort. A total of 13 714 women participated in the baseline survey. The recruited participants were generally representative of women the same age in the Australian population. This analysis included participants who completed survey one in 1996 through to survey six in 2010, when aged 59–64. The survey waves were conducted at approximately three‐year intervals. Women who answered at least one survey between survey two and survey six (*n* = 10 011) were considered for inclusion. Women who were retired at the baseline survey (*n* = 65) or who were unemployed/not in labour force at the baseline survey (*n* = 3018) were not included. Women with hormone replacement therapy (HRT) use or surgical menopause were retained because they may still experience significant VMS and excluding them would reduce representativeness and introduce bias [27, 28]. The final analysis sample included 6928 participants (Figure 1).

### 2.2 Assessment of Women's Retirement Status

Retirement status was captured based on self‐reported measures. In each survey wave, women were asked whether they had experienced retirement either within the past 12 months or more than 12 months ago, with the response options being yes or no. From survey four onwards, an additional question was included to capture respondents' self‐perceived retirement status: “Do you consider yourself to be completely, partially or not retired from the paid workforce?” with the response options being not retired at all, partially retired, completely retired from paid work, gave up paid work more than 20 years ago, or never had paid work. For this study, respondents were classified as retired if they reported having retired (either in the past 12 months or more than 12 months ago) and indicated that they were completely retired from paid work.

### 2.3 Assessment of the VMS Exposures

Women were asked about their separate VMS at each survey using the question: “In the last 12 months, have you had any of the following: hot flushes; night sweats?” The possible responses were measured using the 4‐category Likert scale (never, rarely, sometimes, or often). The responses were dichotomised, with never or rarely indicating the absence of symptoms and sometimes or often indicating the presence of symptoms, based on a previous study conducted in the ALSWH. These dichotomised responses were categorised into four groups: No symptoms—those who reported both hot flushes and night sweats as never or rarely; night sweats only—those who experienced night sweats sometimes or often, but reported hot flushes as never or rarely; hot flushes only—those who experienced hot flushes sometimes or often, but reported night sweats as never or rarely; and both hot flushes or night sweats—those who reported both symptoms as sometimes or often.

### 2.4 Assessments of Covariates

The study accounted for a range of covariates that were identified a priori as having potential association with both VMS and workplace outcomes [12, 23, 29]. These include sociodemographic characteristics such as age (in years), marital status (single, defacto or married, and divorced or widowed or separated), and area of residence (major city of Australia, inner regional, outer regional/remote). Women were asked how well they managed on their available income, with the following response options: impossible or difficult all the time, difficult sometimes, not too bad and easy. A time interval variable was created based on each survey wave. The variable was coded from 0 to 5: Interval 0 (when the women were 45–50 years), Interval 1 (47–52 years), Interval 2 (50–55 years), Interval 3 (53–58 years), Interval 4 (56–61 years), and Interval 5 (59–64 years), which correspond to surveys one through six, respectively. Body Mass Index (BMI) was calculated from self‐reported height and weight (kg/m^2^) at each survey and categorised according to the World Health Organization classification. Women were asked about their smoking habits in each survey and were categorised as never smokers, ex‐smokers, or current smokers. Alcohol consumption was recorded at each survey, except survey three. For survey three, missing alcohol data were imputed by carrying values forward from survey two or backward from survey four. Alcohol intake was categorised as non‐drinker or rarely, low‐risk drinker, risky drinker or high‐risk drinker. In surveys one and two, women were asked whether they had ever been diagnosed with or treated for diabetes mellitus. In subsequent surveys, they were asked whether they had been diagnosed with diabetes mellitus in the past 3 years. Women were classified as having diabetes if they reported a diagnosis or treatment by a doctor. The type of diabetes (type one or type two) was not distinguished in surveys one and four to six; therefore, both types were combined and are referred to as diabetes. Self‐reported data for diabetes has been previously validated. Similarly, in surveys one and two, women were asked whether they had ever been diagnosed with or treated for heart disease, including heart attack or angina. In subsequent surveys, they were asked if they had been diagnosed with heart disease within the past 3 years. Women were classified as having heart disease if they reported a diagnosis or treatment by a doctor. For both diabetes and heart disease, women were coded as not having the condition until the first survey at which it was reported and were then assumed to have the condition at all subsequent surveys.

Perceived stress was measured using the 11‐item Perceived Stress Scale, with mean scores ranging from 0 to 4. Stress levels were categorised as not or somewhat stressed (0–0.99), moderately stressed (1–1.99), and very or extremely stressed (2–4). In each survey, women's menopausal status was determined based on responses to survey questions about menstrual patterns, hormonal use, and having had hysterectomy or oophorectomy. Those who reported having oophorectomy, hysterectomy, or both were classified as having had surgical menopause. Women were classified as premenopausal if they had menstruated in the last 3 months and reported no change in menstrual frequency in the last 12 months. If they reported changes in patterns of menstrual frequency or three to 11 months of amenorrhoea (absence of menstruation), they were classified as perimenopausal, and women were classified as naturally postmenopausal if they reported amenorrhoea for 12 consecutive months or more. If they reported use of oral contraceptives or hormonal supplements, their menopausal status was defined as unclassifiable. Finally, the women were also asked if they were currently using menopausal hormonal replacement therapy (HRT) and the responses were categorised as yes or no.

### 2.5 Statistical Analysis

We used discrete‐time survival analysis using logistic regression to assess the association between VMS and odds of retirement. This approach was selected to account for uncertainty in the timing of retirement events. The findings were presented with odds ratios (OR) and 95% confidence intervals (CIs), with ORs > 1 indicating increased odds of retirement. The models included discrete time interval and time‐varying covariates, with VMS as the main exposure. Model 1 was a univariate model with the predictor variable (VMS), outcome (retirement), and a discrete measure of time interval (survey wave). Covariates were added in steps: Model 2 added area of residence, income and marital status; Model 3 added BMI, smoking status, alcohol intake and stress; Model 4 added HRT, diabetes, heart disease and menopausal status. Statistical analyses were performed using Stata software (version 18.0; Stata Corp).

Participants were followed from baseline until they either reported retirement or were censored at their last observed survey. For women who transitioned to retirement during follow‐up, all observations up to and including the survey in which retirement was first reported were retained and subsequent surveys were excluded as these participants were no longer at risk of the event. To address interval censoring where the exact timing of retirement was unknown but fell between two survey points, the timing of retirement was assumed to be between the last survey in which the participant was working and the survey in which retirement was first reported. Discrete‐time survival models handle such interval censoring [30, 31]. To model this interval‐censored transition, the retirement indicator was shifted back by one survey, and the final post‐retirement observation was removed. Logistic regression was then applied to model the conditional probability of event occurrence at each time point, given that the individual had not yet experienced the event. The study excluded observations with missing data on the outcome using list‐wise deletion. However, all covariates had less than 5% missing data and were therefore retained in the analysis.

## 3 Results

### 3.1 Study Participant Characteristics

Table 1 provides an overview of the participants' socio‐demographic details and health risk factors at baseline (aged 45–50). The women were a mean age of 47.6 (±1.5 SD) years, and the majority lived in a major city (39%), followed by inner regional areas (37.7%), and were married or in a de facto relationship (82.6%). More than half (53.8%) had never smoked, and (52.9%) were of a healthy weight. The majority (61.9%) indicated that their ability to manage on their income was easy or not too bad. Over half of the participants (50.8%) were employed full‐time, while 47.6% worked part‐time. The proportions of women reporting no VMS, only hot flushes, only night sweats, and both hot flushes and night sweats in survey one were 66.2%, 11.2%, 3.6%, and 18.3%, respectively (Table 1). The proportion of VMS varied over time, with fewer women reporting no symptoms declining most noticeably at ages 50–55 and more reporting both hot flushes and night sweats than the individual symptoms alone (Figure S1). Retirement patterns showed a clear age‐related increase across the cohort. By survey 2, less than 1% of women had retired (ages 45–50), with this gradually increasing to 38.5% by survey 6 (ages 59–64) (Figure S2). Women's work patterns prior to retirement revealed diverse pathways. Among women who retired during follow‐up, approximately 35% retired directly from full‐time work, while 26% retired directly from part‐time work. Nearly 19% of women reduced their hours from full‐time to part‐time prior to retirement. Smaller proportions exited via unemployment before retirement, including 7% who transitioned from full‐time to unemployed and 13% from part‐time to unemployed. Only 1% followed less common transitions, such as moving from part‐time back to full‐time before retirement (Figure S3).

**TABLE 1:** Baseline characteristics of women in the 1946–1951 cohort of the Australian Longitudinal Study on Women's Health (age 45–50 years, *n* = 6928).

Women's characteristics	Frequency (%)
Age, mean ± SD	47.6 (1.5)
Area of residence, *n* (%)	
Major cities	2704 (39.0)
Inner regional	2612 (37.7)
Outer regional/remote	1612 (23.3)
Marital status, *n* (%)^a^	
Single	224 (3.2)
Married or de facto	5723 (82.6)
Separated/widowed/divorced	956 (13.8)
Smoking status, *n* (%)^a^	
Never smoker	3725 (53.8)
Ex‐smoker	1978 (28.6)
Current smoker	1027 (14.8)
Ability to manage on income, *n* (%)^a^	
Impossible/difficult always	741 (10.7)
Sometimes difficult	1874 (27.0)
Easy/not too bad	4286 (61.9)
Body mass index, *n* (%)^a^	
Underweight (< 18.5 kg/m^2^)	111 (1.7)
Healthy weight (18.5–24.9 kg/m^2^)	3668 (52.9)
Overweight (25.0–29.9 kg/m^2^)	1879 (27.1)
Obese (≥ 30.0 kg/m^2^)	1042 (15.0)
Stress (scores), *n* (%)^a^	
Not/somewhat stressed	4594 (66.3)
Moderately stressed	1864 (26.9)
Extremely stressed	293 (4.2)
Alcohol intake, *n* (%)^a^	
Non/rarely drinks	2735 (39.5)
Low risk drinker	3787 (54.7)
Risky/high risk drinker	357 (5.2)
Menopausal status, *n* (%)^a^	
Premenopausal	2567 (37.0)
Perimenopausal	1545 (22.3)
Postmenopausal	317 (4.6)
Surgical menopause	1472 (21.2)
Hormonal use	1014 (14.6)
Use of HRT, *n* (%)^a^	
Yes	1194 (17.2)
No	5721 (82.6)
Vasomotor symptoms, *n* (%)^a^	
No	4590 (66.2)
Only night sweats	248 (3.6)
Only hot flushes	777 (11.2)
Both hot flushes and night sweats	1270 (18.3)
Diabetes, *n* (%)^a^	
Yes	124 (1.8)
No	6782 (97.9)
Heart disease, *n* (%)^a^	
Yes	110 (1.6)
No	6779 (97.8)
Working hours, *n* (%)^a^	
Part‐time worker	3295 (47.6)
Full‐time worker	3520 (50.8)

*Note:* Area of residence, *n* = 21; marital status, *n* = 25; smoking status, *n* = 198; body mass index, *n* = 228; ability to manage on income, *n* = 27; VMS, *n* = 43; alcohol intake, *n* = 49; HRT, *n* = 13; menopausal status, *n* = 13; stress (scores) *n* = 177; diabetes, *n* = 22, heart disease, *n* = 39, workhours, *n* = 113.

^a^ The number of missing values.

### 3.2 Association of VMS of Menopause With Retirement

The presence of both hot flushes and night sweats was associated with a modest but statistically significant increase in the odds of retirement, both in unadjusted and adjusted models (OR = 1.17, 95% CI: 1.07, 1.28; AOR = 1.13, 95% CI: 1.03, 1.25) (Table 2). In contrast, experiencing only night sweats was not significantly associated with retirement in either the unadjusted or adjusted analyses (OR = 1.17, 95% CI: 0.94, 1.46; AOR = 1.19, 95% CI: 0.94, 1.50). Similarly, experiencing only hot flushes was not significantly associated with increased odds of retirement (OR = 1.00, 95% CI: 0.89, 1.14; AOR = 0.97, 95% CI: 0.85, 1.11). These findings suggest that the combined effects of both symptoms may have a stronger association with women's decisions to exit the workforce (retirement), compared to experiencing either symptom alone (Table 2).

**TABLE 2:** The association between vasomotor symptoms of menopause and retirement among 6928 women: Odds ratios (OR) and their 95% confidence interval (CI).

Exposure	Model 1, OR	Model 2, AOR	Model 3, AOR	Model 4, AOR
	(95% CI)	(95% CI)	(95% CI)	(95% CI)
Vasomotor symptoms				
No	1.0	1.0	1.0	1.0
Only night sweats	1.17 (0.94, 1.46)	1.13 (0.89, 1.42)	1.16 (0.92, 1.46)	1.19 (0.94, 1.50)
Only hot flushes	1.00 (0.89, 1.14)	0.98 (0.86, 1.12)	0.99 (0.87, 1.13)	0.97 (0.85, 1.11)
Both hot flushes and night sweats	1.17 (1.07, 1.28)*	1.13 (1.03, 1.24)*	1.12 (1.01, 1.23)*	1.13 (1.03, 1.25)*
Time interval (age)				
Interval 1 (47–52 years)	1.0	1.0	1.0	1.0
Interval 2 (50–55 years)	9.05 (6.58, 12.45)*	9.50 (6.86, 13.17)*	9.12 (6.5, 12.79)*	8.74 (6.14, 12.44)*
Interval 3 (53–58 years)	18.49 (13.61, 25.12)*	19.61 (14.33, 26.83)*	18.28 (13.19, 25.33)*	15.85 (11.22, 22.39)*
Interval 4 (56–61 years)	22.79 (16.78, 30.94)*	24.34 (17.84, 33.37)*	23.65 (17.09, 35.74)*	19.21 (13.57, 27.18)*
Interval 5 (59–64 years)	31.14 (22.95,4 2.25)*	33.83 (24.75, 46.24)*	31.58 (22.83, 43.69)*	24.53 (17.31, 34.73)*
Area of residence				
Major cities		1.0	1.0	1.0
Inner regional		1.02 (0.93, 1.12)	1.02 (0.93, 1.13)	1.03 (0.94, 1.14)
Outer regional/remote		0.93 (0.83, 1.04)	0.91 (0.81, 1.02)	0.93 (0.84, 1.05)
Marital status				
Married/de facto		1.0	1.0	1.0
Single		0.59 (0.46, 0.78)*	0.59 (0.45, 0.78)*	0.61 (0.46, 0.81)*
Separated/Divorced/Widowed		0.55 (0.48, 0.62)*	0.52 (0.45, 0.59)*	0.51 (0.45, 0.58)*
Ability to manage on income				
Impossible/difficult always		1.49 (1.31, 1.70)*	1.40 (1.21, 1.62)*	1.26 (1.08, 1.46)*
Sometimes difficult		0.98 (0.89, 1.08)	0.95 (0.85, 1.06)	0.91 (0.82, 1.02)
Easy/not too bad		1.0	1.0	1.0
BMI				
Healthy weight			1.0	1.0
Underweight			1.67 (1.14, 2.44)*	1.51 (1.03, 2.22)*
Overweight			1.14 (1.04, 1.26)*	1.13 (1.02, 1.25)*
Obese			1.27 (1.14, 1.42)*	1.21 (1.08, 1.34)*
Smoking status				
Never smoker			1.0	1.0
Ex‐smoker			0.95 (0.86, 1.05)	0.93 (0.85, 1.03)
Current smoker			1.06 (0.92, 1.22)	0.97 (0.84, 1.11)
Stress (scores)				
Not/somewhat stressed			1.0	1.0
Moderately stressed			0.99 (0.89, 1.11)	0.95 (0.86, 1.06)
Extremely stressed			1.03 (0.80, 1.33)	0.86 (0.67, 1.12)
Alcohol intake				
Low risk drinker			1.0	1.0
Non/rarely drinker			1.22 (1.02, 1.23)	1.06 (0.97, 1.17)
Risky/high risk drinker			1.19 (1.01, 1.41)*	1.22 (1.03, 1.44)*
Menopausal status				
Perimenopausal				1.0
Premenopausal				0.81 (0.53, 1.23)
Postmenopausal				1.49 (1.21, 1.84)*
Surgical menopause				1.72 (1.39, 2.15)*
Hormonal use				1.14 (0.86, 1.51)
HRT use				
Yes				1.13 (0.97, 1.28)
No				1.0
Diabetes				
Yes				1.37 (1.13, 1.66)*
No				1.0
Heart disease				
Yes				1.39 (1.01, 1.75)*
No				1.0

* Indicates statistical significance less than 0.05. Intervals 0 to 5 (ages 45–64) correspond to surveys one to six. Interval 0 (45–50 years) is not reported in the table, as women who had retired in survey one were excluded. OR > 1 indicates higher odds of retirement, whereas an OR < 1 indicates lower odds of retirement. Model 1: A univariate model with the exposure variable, outcome, and a discrete measure of time (age). Model 4: Model 1 plus area of residence, income, marital status, smoking status, BMI, alcohol intake, stress, diabetes, heart disease, HRT and menopausal status.

### 3.3 Covariates Associated With Retirement

Aside from VMS, our analysis identified additional health and sociodemographic factors significantly associated with retirement. Table 2 shows associations between the included covariates and women's reports of retirement. Taking these covariates into account alongside VMS offers a broader context for understanding women's retirement from the workforce. Notably, some of these factors demonstrated stronger associations with retirement than VMS. Time interval (age) was unsurprisingly one of the strongest factors associated with retirement. Compared to women in interval 1 (ages 47–52), those in interval 5 (ages 59–64) had significantly higher odds of retirement. Similarly, compared to women who reported that managing on their income was easy or not too bad, those who reported it was impossible or difficult always had significantly higher odds of retirement. BMI was also an important factor, with both underweight, overweight, and obese women being more likely to experience retirement compared to women of healthy weight. Ill health also appeared to be an important factor influencing retirement. For example, women with diabetes or heart disease had significantly higher odds of retirement than those without the condition. Women who had undergone surgical menopause, as well as those who were postmenopausal, were more likely to retire compared to premenopausal. In contrast, the use of HRT did not show a significant association with retirement (Table 2).

## 4 Discussion

### 4.1 Main Findings

To the best of our knowledge, this is the first study that examines the association between VMS and retirement using a large, nationally representative longitudinal dataset. Overall, the findings suggest that women who experienced both hot flushes and night sweats reported higher odds of retirement. However, neither hot flushes nor night sweats alone were found to be significantly associated with retirement. These associations were independent of potential covariates. This finding provides a unique contribution to the literature by highlighting the association of VMS and retirement. By focusing on retirement as a workforce outcome using longitudinal data, our study differs from previous studies that assessed workforce participation using other adverse work outcomes such as reduced work productivity, work absenteeism, and poor work performance [13, 16, 22, 23]. In addition to VMS of menopause, other factors including diabetes, heart disease, marital status, BMI, ability to manage on available income, and age (time interval) were strongly associated with retirement.

### 4.2 Interpretation

Our findings indicate that women who experienced both hot flushes and night sweats reported higher odds of retirement compared to those with no VMS. There are few other studies to directly compare the findings of an association between VMS and retirement. However, some other studies can support our findings. For example, a cross‐sectional study done in the USA indicated that women experiencing menopausal symptoms including VMS, sleep disturbances, joint pain, and mood changes reported adverse work outcomes such retirement or changing jobs [32]. A longitudinal study by Evandrou et al. [16] also reported that, compared to women with no symptoms, those experiencing menopausal symptoms such as VMS, sleep disturbances, joint pain, and others reported a higher proportion of retirement or reduction in their working hours. Likewise, a cross‐sectional study of female hospital workers in Ireland examined the association between menopausal symptoms, including VMS, fatigue, poor memory, depression, and work‐related outcomes. Among participants reporting these symptoms, 7% had changed roles, 8% had reduced their working hours, 35% indicated that symptoms influenced their career decisions, and 2% had resigned from their jobs compared with women without symptoms [14]. The observed associations might be attributed to the disruptive nature of menopausal symptoms, which have been shown to negatively affect sleep quality, mood and overall wellbeing. These psychological and physical challenges may influence the decision to leave the workforce [9, 33]. Another possible explanation is that women with frequent VMS are at increased risk of metabolic diseases, including diabetes, and cardiovascular disease, which may contribute to exit from the workforce [9, 10, 34]. Although women with diabetes and heart disease had higher odds of retirement, our analyses indicate that VMS remained independently associated with workforce exit after adjusting for these comorbidities. In contrast, Hardy et al. [22] reported that VMS were not directly associated with retirement. However, experiencing severe hot flushes at work predicted women's intention to exit the workforce. Hardy et al. study's findings may not be representative of the general population due to its small sample size (*n* = 216).

VMS have been previously associated with reduced work productivity and increased work absenteeism [13, 23, 35]. At the same time, evidence from systematic review shows that the link between VMS and work outcomes is not straightforward, with findings mixed and often confounded by other factors [36]. This suggests that VMS affect women differently, and for some may contribute to reduced working hours, leaving employment, or early retirement [16, 37].

Studying retirement as an outcome is therefore particularly relevant, as it captures the impact of VMS on women's economic, social and psychological wellbeing [38, 39]. Importantly, not all women experiencing VMS report retirement [38]. The observed association should be considered within the Australian retirement context (1996–2010), when women commonly retired in their late 50s to early 60s and the Age Pension eligibility age was 60 years [40, 41].

We found that sociodemographic and health‐related factors were also associated with the odds of retirement. As expected, age had a very strong association with retirement. In our study, compared to married women, those who were never married or who were separated, divorced, or widowed were less likely to be retired. This finding was consistent with previous research, which indicated that marital status affects retirement decisions, with being married associated with a greater likelihood of early retirement [42, 43]. This may be because married women often have greater financial security through shared household income [42, 43]. In contrast, women in our study who reported that managing on their available income was difficult or impossible had higher odds of retirement, likely reflecting a more constrained pathway in which financial strain, poorer health, and job insecurity may commonly be associated with involuntary exit from the work force for various reasons [44]. We did not find an association between HRT use and retirement. Even though HRT use may reduce vasomotor symptoms, retirement decisions are driven by broader factors [44].

Our findings also revealed that BMI is one of the factors associated with retirement. This is consistent with other studies, which reported that body weight outside the recommended range is a risk factor for retirement. The possible explanation might be that both overweight and obesity can contribute to the early onset of chronic conditions and disabilities, which in turn may lead to frequent work absenteeism, loss of work productivity and limitations in daily functioning, ultimately resulting in retirement or exit from the workforce. Women who experienced ill health, including diabetes and heart disease, had higher odds of retirement than those without these conditions. This finding is consistent with other studies reporting that individuals with chronic conditions such as diabetes and heart disease are more likely to retire early [21, 34, 45].

In our study, postmenopausal women were more likely to be retired than perimenopausal women. In contrast, an Australian study reported that postmenopausal women had lower intention to leave their organisations than pre and perimenopausal women. This discrepancy might be due to a smaller sample size (*n* = 1092) and the lack of control for important covariates such as age. Similarly, Hardy et al. [46] reported no difference in intention to exit the workforce between pre, peri, and postmenopausal women [22].

The potential associations between VMS and retirement carry significant psychological, financial, and social consequences for women and their families [47]. For instance, VMS can lead to anxiety and depression, which affect both work and the overall well‐being of women [48, 49]. Additionally, the potential financial strain from reduced household income following retirement may exacerbate these challenges [35]. These impacts extend beyond the individual, affecting society as a whole, as midlife women are an experienced, reliable, and loyal segment of the workforce [12, 50]. To mitigate these effects and retain a skilled workforce during the menopausal years, a collaborative approach is needed [12]. Employers should foster supportive workplaces [51], including flexible work arrangements, providing menopause‐related training, and encouraging open communication [51, 52]. At the same time, workplace support must be carefully designed to protect privacy [52, 53]. This may also help to avoid further stigmatizing women in the workforce [54]. In addition, health care providers should also treat menopause symptoms to promote the overall health of women [55].

### 4.3 Strengths and Limitations

Our study had a number of strengths and limitations. The key strength includes the use of a large, population‐based sample of mid‐aged women from a national longitudinal cohort study, the longitudinal analysis and the inclusion of potential time‐varying covariates. The study also used a novel design and methodology to examine the association between VMS and retirement among midlife women using discrete‐time survival analysis. We used self‐reported data to capture retirement status, which may introduce information bias. In particular, the wording of the retirement question may not clearly distinguish between individuals who voluntarily retire and those who stop working due to health problems, thereby introducing a risk of misclassification. An additional limitation is that the data were collected between 1996 and 2010, when hormonal therapies and patterns of work and retirement differed from today, which may limit generalisability. However, long‐term studies tracking women through menopause and into retirement are rare, so these historical data still offer valuable insight into the relationship between VMS and retirement. We note that these associations cannot imply causality.

## 5 Conclusion

In this study, women who experienced both hot flushes and night sweats reported higher odds of retirement, suggesting that the combined effect of VMS may affect women's ability or decision to remain in the workforce. Other factors, including overweight, surgical menopause, income, and chronic conditions such as diabetes and heart disease, may also contribute to retirement. Improving access to effective symptom management, along with examining how workplace conditions shape women's experiences of menopause, may help guide strategies to support continued workforce participation in midlife. Further research is needed to validate these associations and to assess the moderating role of health‐related factors in the relationship between VMS and retirement.

## Author Contributions

B.W.A., M.W., and L.R.T. collaboratively designed the study. B.W.A. conducted the data analysis and drafted the manuscript. L.R.T. and M.W. critically reviewed and provided revisions to the manuscript. All authors have read and approved the final version of the manuscript.

## Funding

B.W.A. was granted the University of Queensland Research Training Stipend Scholarship to pursue his PhD. The ALSWH is funded by the Australian Government Department of Health Disability and Ageing. The funder had no role in study design and conceptualisation, data collection, data analysis, data interpretation or writing of the manuscript and no role in the decision to submit the manuscript for publication.

## Ethics Statement

The Australian Longitudinal Study on Women's Health (ALSWH) received ethical approval from the Human Research Ethics Committees at both the University of Queensland and the University of Newcastle. Informed consent was obtained from all participants prior to the completion of each survey. Ethical approval for the 1946–1951 cohort was granted by the University of Newcastle (H‐076‐0795) and the University of Queensland (2004/HE000224).

## Conflicts of Interest

The authors declare no conflicts of interest.

## Supporting information


**Figure S1:** Proportion of VMS in each survey.

**Figure S2:** Proportion of retired women across surveys.

**Figure S3:** Workforce participation patterns preceding retirement among women who retired during follow‐up.

## Data Availability

ALSWH survey data are owned by the Australian Government Department of Health, Disability and Ageing, and due to the personal nature of the data collected, release by ALSWH is subject to strict contractual and ethical restrictions. De‐identified data are available to collaborating researchers where a formal request to make use of the material has been approved by the ALSWH Data Access Committee. The committee is receptive to requests for datasets required to replicate results. Information on applying for ALSWH data is available from https://alswh.org.au/for‐data‐users/applying‐for‐data/.
