# Supplementary material for: Association Between Vasomotor Symptoms of Menopause and Retirement: A Prospective Longitudinal Study
Source: BJOG. 2026 May 12;133(11):2044–53. doi: 10.1111/1471-0528.70264 (PMC13571884; doi:10.1111/1471-0528.70264)
Supplement: Supplementary file 1 [file BJO-133-2044-s001.docx]

Proportion of VMS

| Survey/year | No symptoms | Only night sweats | Only hot flashes | Both hot flashes and night sweats |
| --- | --- | --- | --- | --- |
| s1(45-50) | 66.7 | 3.6 | 11.3 | 18.4 |
| s2(47-52) | 59.1 | 3.7 | 12.1 | 25.2 |
| s3(50-55) | 45.1 | 2.7 | 12.4 | 39.7 |
| s4(53-58) | 48.9 | 2.7 | 15 | 33.4 |
| s5(56-61) | 53.9 | 2.9 | 13.8 | 29.4 |
| s6(59-64) | 63.1 | 3.5 | 10.9 | 22.5 |

Figure S1. Proportion of VMS in each survey
