# Supplementary material for: Association Between Vasomotor Symptoms of Menopause and Retirement: A Prospective Longitudinal Study
Source: BJOG. 2026 May 12;133(11):2044–53. doi: 10.1111/1471-0528.70264 (PMC13571884; doi:10.1111/1471-0528.70264)
Supplement: Supplementary file 4 [file BJO-133-2044-s002.docx]

Figure (S2). Proportion of retired women across surveys
